# Synthesis and Characterization of Chitosan Nanoaggregates from Gladius of* Uroteuthis duvauceli*

**DOI:** 10.1155/2016/5379424

**Published:** 2016-02-10

**Authors:** J. R. Anusha, Albin T. Fleming

**Affiliations:** Department of Advanced Zoology and Biotechnology, Loyola College, Chennai, Tamil Nadu 600 034, India

## Abstract

We report the synthesis, characterization, and biological properties of chitosan nanoaggregates from gladius of squid,* Uroteuthis duvauceli*. *β*-Chitin extracted from gladius was deacetylated to chitosan and further reduced to nanosize using ionic gelation process. The morphology and occurrence of chitosan nanoaggregates (CSNA) were observed using transmission electron microscopy (TEM). The degree of deacetylation (DD%) calculated from Fourier transform infrared (FTIR) spectrum showed high value (~94 ± 1.25%) for chitosan. The CSNA depicts low molecular weight, stable positive zeta potential, and less ash and moisture content with high water and fat binding capacity. The antimicrobial activity was tested against pathogenic microorganisms, which depicted significant rate of inhibition against* Staphylococcus aureus* and* Escherichia coli* due to high cellular uptake. The antioxidant analysis for CSNA demonstrated high reducing power and scavenging activity towards superoxide radicals compared with the commercially available chitosan. Furthermore, nanoaggregates exhibited low cytotoxic behavior in biological* in vitro* tests performed using cervical cancer cell line. These results indicate that chitosan nanoaggregates synthesized from waste gladius will be highly efficient and safe candidate for biological applications as food packing film, drug carrier, and tissue engineering.

## 1. Introduction

In recent years, the biopolymers have been considered as potential eco-friendly substitute for the use of nonbiodegradable and renewable materials. One among such naturally abundant biopolymers is chitin, a mucopolysaccharide formed of 2-acetamido-2-deoxy-*β*-D-glucose through *β* (1 → 4) linkages. Depending on the source, chitin exists in three different polymorphic forms: *α*, *β*, and *γ*. Rhombic *α*-chitin resembles chain-like structure arranged in an antiparallel direction with strong intermolecular hydrogen bonds found in shells of crabs, shrimps, and other arthropods [1, 2]. The pure form of monoclinic *β*-chitin is patterned in parallel direction with weak intermolecular hydrogen bonds occurring in gladius of squid fish commonly known as squid pen [3]. *γ*-Chitin is extracted from fungal microorganisms which is a combination of *α*- and *β*-chitin [4]. Chitin is a hard, inelastic material which is insoluble in most of the solvents due to its compact structure [5]. Hence, the acetyl groups were removed from chitin through deacetylation process, to form a deacetylated derivative called chitosan [6]. The structure of chitosan has N-atom with unshared pair of electrons that can potentially be donated, and amino groups are mostly protonated with no possibility of donating electrons. In solution, chitosan has to be acidified in order to dissolve the polymer; p*K*_a_ of chitosan is ~6.3. It has attracted considerable interest in the field of research owing to excellent biodegradability, biocompatibility, and bioactive and nontoxic nature [7, 8]. Due to its promising properties, now it is used in vast range of applications such as waste water treatment, additives for cosmetics, fibres for textiles, photographic papers, biodegradable films, biomedical devices, and microcapsule implants for controlled release in drug delivery [9–11].

With the aid of nanotechnology, nanosized biopolymers with enhanced properties have been considered as a promising option in the field of food packing, drug delivery, biosensors, and other biomedical applications [12–14]. Nanoparticles synthesized using chemical as well as mechanical method exhibit much improved properties compared with normal sized biopolymers due to high aspect ratio and surface area. Moreover, these nanoparticles with high antimicrobial profile have been devoted for the development of food packaging films in addition to improved mechanical barrier, rheological and thermal properties [15–18]. From the recent literatures, researchers have clearly demonstrated the low or nontoxic activity of chitosan nanoparticles with different chemical modifications [19–21]. In addition, chitosan scaffolds have been used in tissue engineering for skin repair and wound healing with regeneration mechanism [22]. Moreover, the mucoadhesive character facilitates the administration of poorly absorbable drugs as well as macromolecules such as nucleic acids, growth factors, and antigens across epithelial barriers [21, 23]. It was reported that the chitosan/polyguluronate nanoparticles with low cytotoxicity were used for the effective delivery of siRNA to HEK 293FT and HeLa cells [24].

For the commercial production of chitosan, chitin from shrimp and crab shells was used as raw material, which required high production cost and multiple chemical processes such as demineralization, deproteinization, and decolourization [25]. Gladius of squid is another rich source of chitosan, which is a transparent material thrown as waste from seafood processing industries. Chitosan production from gladius is cost-effective and prevents the usage of excess acids and alkaline pollutants due to its low impurities and absence of coloured compounds. In addition, it shows better reactivity, solubility, and swelling than from other sources due to much weaker molecular hydrogen bonding [7]. Most frequently, nanoparticles were synthesized according to bottom-up approach as a result of self-assembling or cross-linking process [26].

From the above-mentioned application, it is evident that the chitosan and its nanoparticles were highly effective in biomedical applications. Meanwhile, a well suitable, abundant source and easy processing steps are necessary for chitosan nanoparticle synthesis. In the present study, the chitosan nanoaggregate has been extracted from the gladius of squid,* Uroteuthis duvauceli*. The gladius was subjected to deproteinization and demineralization to form *β*-chitin and further deacetylated to chitosan. The nanoaggregate of chitosan was synthesized by ionic gelation process using tripolyphosphate solution. The physical and chemical properties of as-prepared chitosan nanoaggregate were analyzed and discussed in detail. In addition, the antimicrobial, antioxidant, and cytotoxic activities were evaluated to identify bioactive potential of synthesized nanostructure.

## 2. Experimental

### 2.1. Materials

Commercially available chitosan (medium molecular weight, 190 to 300 kDa, DD, 75 to 85%) and sodium tripolyphosphate were purchased from Sigma Aldrich, Korea. The chemicals for biological assays were from Himedia (Mumbai). All other chemicals were of analytical grade and used without further purification. The squid,* Uroteuthis duvauceli*, was from the coastal region of Kanyakumari District, Tamil Nadu, India. The gladius was separated from fish and sealed in plastic bags and stored in 4°C before further analysis.

### 2.2. Isolation of *β*-Chitin

The gladius collected from squids (*U. duvauceli*) was cleaned to remove muscle debris and washed thoroughly with water to remove adherent proteins and soluble organic materials. The cleaned gladius was dried at room temperature and pulverised using mortar and pestle. *β*-Chitin was extracted from powdered gladius by sequence of processes such as deproteinization using 1 M NaOH at 50°C for 5 h and demineralization in 1 M HCl at room temperature for 2 h under constant stirring. The extract was filtered and washed with deionized (DI) water until it attains neutral pH followed by dehydration using methanol and acetone. Finally, the obtained chitin powder was dried overnight in a vacuum oven at 60°C. As-prepared chitin powder was subjected to deacetylation in 50% of 1 M NaOH solution at 60°C under nitrogen atmosphere for 6 h. The obtained white chitosan precipitate was washed several times with DI water to attain neutral pH.

### 2.3. Synthesis of Chitosan Nanoaggregate (CSNA)

**Scheme 1:**
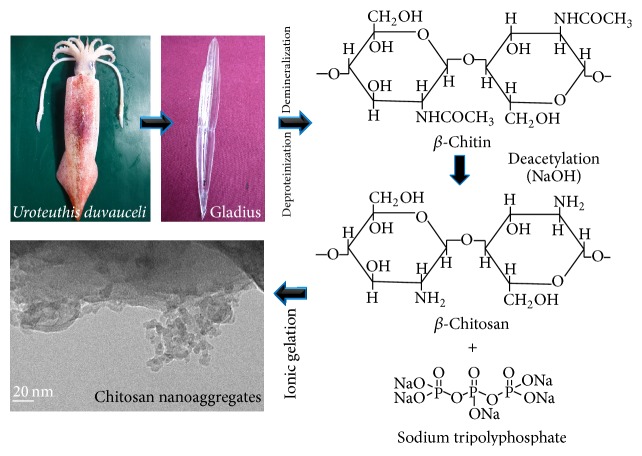
Illustrating the steps involved in the synthesis of chitosan nanoaggregates from gladius of squid,* Uroteuthis duvauceli*.

As-prepared chitosan particles were deduced to nanosized particles by ionic gelation process using sodium tripolyphosphate. The 100 mL of chitosan solution was added to 40 mL of TPP (1.0 g/L) solution, stirred for 2 h at an ambient temperature to form optimized quantity of opalescent solution. The opalescent solution was centrifuged at 10,000 rpm and the pellets were rinsed with DI water, dried, and used for further analysis (Scheme 1).

### 2.4. Physicochemical Characterization

The morphology of CSNA was recorded using transmission electron microscopy (TEM, JEOL model JEM 2011). The Fourier transform infrared (FTIR) spectra of CSNA were recorded using Perkin Elmer US/Spectrum GX spectrometer by KBr pellet technique. The degree of deacetylation (DD%) and functional groups were evaluated from FTIR absorbance peak of Std.CS and CSNA at 1655 cm^−1^ for amide I and at 3450 cm^−1^ for OH group. The DD% was calculated using the modified baseline technique [27] as shown in

(1) $$\begin{array}{c} DD\%=\left(\;\frac{A_{1655}}{A_{3450}}\;\right)\times 115, \end{array}$$

where *A*_1655_ and *A*_3450_ are the absorbance at 1655 cm^−1^ and 3450 cm^−1^, respectively.

XRD patterns of CSNA were obtained with X-ray diffractometer (D/MAX-2200, Rigaku Co., Japan) using Cu K*α* radiation with a wavelength of 0.154 nm. The scattering intensities were measured over an angle range from 10 to 60° (2*θ*) with a scanning rate 2°/min.

The average molecular weight was measured from viscosities by Mark-Houwink equation. The samples were dissolved in 0.17 M (1%, v/v) acetic acid, filtered through a sinter glass, and the viscosity of solution was measured with Ubbelohde-type capillary viscometer at 25 ± 1°C. Then, *M*_*v*_ was calculated using Mark-Houwink equation:

(2) $$\begin{array}{c} \left[\;\eta \;\right]=K{\left(\;M_{v}\;\right)}^{\alpha }, \end{array}$$

where [*η*] and *M*_*v*_ represented intrinsic viscosity and viscosity molecular weight and *K* and *α* are viscosity constant with literature values 1.81 × 10^−5^ and 0.93, respectively.

In addition, zeta potential of CSNA was analyzed using Zetasizer (Malvern Instruments, UK) based on dynamic light scattering method. The ash and moisture content were estimated as described methods from the Association of Official Analytical Chemistry [28].

For the analysis of water binding capacity (WBC) and fat binding capacity (FBC), 0.5 g of sample was kept in 50 mL centrifuge tube and 10 mL of water or soybean oil was added and mixed thoroughly for 1 min on a vortex mixer. The tubes were incubated under constant shaking for 30 min at room temperature and centrifuged at 2500 rpm for 25 min. The supernatant was decanted and the tube was weighed. From the measurement, WBC and FBC were calculated as follows:

(3) WBC%=water  boundgsample  weightg×100,FBC%=fat  boundgsample  weightg×100.

### 2.5. Antimicrobial Activity

Antimicrobial assay was performed using Kirby-Bauer disk diffusion method. For antibacterial assay, the bacteria were lawn cultured on nutrient agar plates with CSNA loaded disc along with the reference standard antibiotics, ofloxacin, and incubated at 37°C for 24 h.* In vitro* antifungal assay was performed in the surface of Sabouraud dextrose agar (SDA) plates seeded with 0.1 mL of spore suspension (104 spores/mL). Disc loaded with CSNA was placed in SDA plates and incubated at 25°C for 3–5 days. The minimal inhibitory concentrations (MICs) of the CSNA and Std.CS were determined for each antimicrobial activity against selected microorganisms. The zone of growth inhibition was measured in triplicate and the mean ± standard deviation (SD) is recorded.

### 2.6. Antioxidant Assay

The antioxidant capacities of CSNA were determined using reducing power and superoxide radical scavenging assay. The reducing property was quantified by assessing the abilities of chitosan samples to reduce FeCl_3_ solution described by Oyaizu [29]. The superoxide radical scavenging ability of CSNA and Std.CS was also assessed by* in vitro* method reported by Jing and Zhao [30]. The resultant absorbance was measured at 590 nm with UV visible spectrometer (Shimadzu, UV-1800, Japan). The scavenging activity (%) was calculated using

(4) Scavenging  activity%=1−absorbance  of  testabsorbance  of  blank×100.

### 2.7. Cell Toxicity Analysis

HeLa (human cervical carcinoma) cells were used to test the* in vitro* cytotoxicity of CSNA and Std.CS. Cells were seeded in 96-well plates at a density of 50,000 cells/well and cultured in Dulbecco's Modified Eagle Medium (DMEM, Gibco), supplemented with 10% fetal bovine serum (FBS) and penicillin. The culture was incubated overnight at 37°C in 95% humidified air along with 5% CO_2_ environment. After 24 h of postincubation with CSNA and Std.CS, cell proliferation was determined by adding standard 3-(4,5-dimethylthiazol-2-yl)-2,5-diphenyltetrazolium bromide (MTT) to each well. Then, the optical density was measured at 570 nm after 2 h of incubation.

### 2.8. Statistical Analysis

Each experiment was performed in triplicate and the results shown were mean values ± standard deviation (SD) of data obtained. The difference between pairs of means was resolved by confidence intervals using Tukey's tests with the level of significance set at *P* < 0.05. In addition, OriginPro 8 and Microsoft Excel, 2010, programs were used for analysis.

## 3. Results and Discussion

### 3.1. Characterization of Chitosan Nanoaggregate

**Figure 1:**
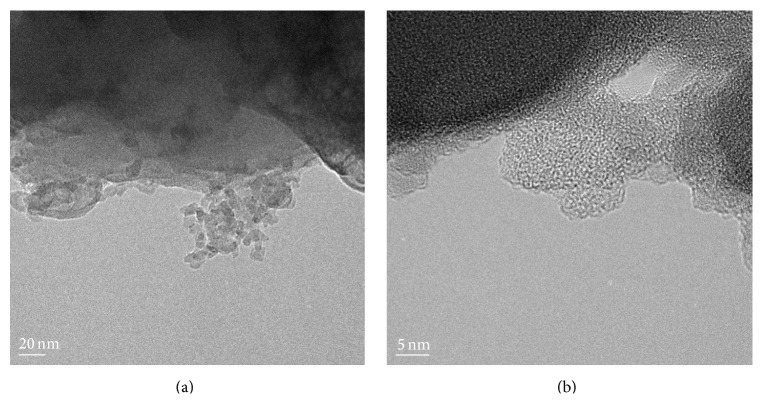
(a) TEM and (b) HRTEM images showing CSNA.

The yield of CS from *β*-chitin isolated from gladius of* U. duvauceli* was 49.28%, which was higher or even comparable with the results of other squid species [31, 32]. The obtained *β*-chitin was reduced to nanosized particles by ionic gelation process using tripolyphosphate. As-prepared CSNA morphology was analyzed by TEM image which showed an agglomeration of chitosan particles of size ≤ 50 nm in the form of nanoaggregates (Figure 1(a)).  Figure 1(b) shows the resultant high resolution (HRTEM) image of CSNA. The image clearly displays the lattice fringes of sample which represents high degree of crystallinity. The chitosan nanoaggregates were formed by the interaction between positively charged amino groups of chitosan and negative charged tripolyphosphate ions at room temperature [26]. Moreover, the gel chitosan was reduced to opalescence while reacting with tripolyphosphate which relied on the formation of inter- and intramolecular cross-linkages medicated by the anionic molecule [33].

**Figure 2:**
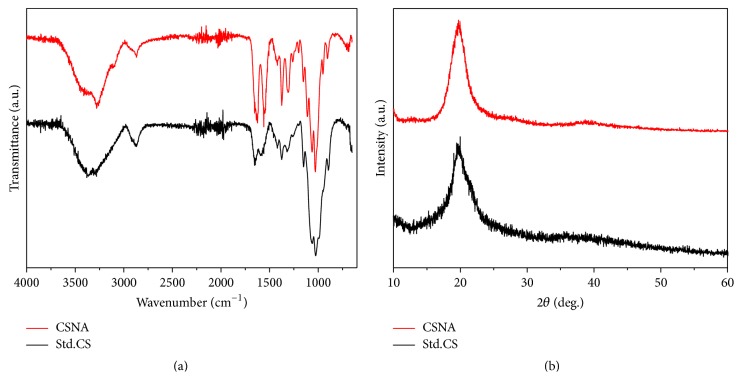
(a) Comparative FTIR spectra and (b) XRD pattern of CSNA and Std.CS.

The functional groups of CSNA and Std.CS were analyzed from the FTIR spectra shown in Figure 2(a). The spectrum for CSNA showed a band at 3273 cm^−1^ which corresponds to NH_2_ and OH stretching. The bands at 2871 cm^−1^ are responsible for aliphatic CH stretching whereas the band at 1561 cm^−1^ was attributed to amide stretching of C=O. Meanwhile, the C-O-C stretching vibrational modes were found at 1064 and 1029 cm^−1^, respectively. Similar functional groups vibration was noticed in Std.CS which well agreed with the previous reports. In CSNA, the peak observed at 1204 cm^−1^ and 890 cm^−1^ for P=O stretching and P-O bending clearly represents the presence of TPP which was relevant to the reported literature [33]. Moreover, NH_2_ absorption peak was found in 1596 cm^−1^ for Std.CS, while in the case of CSNA, the NH_2_ stretch vibration peak slightly drifted to a low wave number, which confirmed that the phosphate group linked to amino group and formed strong intermolecular hydrogen bonds to produce nanoaggregates [26].

The degree of deacetylation depends on the number of glucosamine units of biopolymer chain with respect to the total number of units, which depicts the formation of chitosan from chitin. Moreover, DD% of chitosan could dictate its solubility, swelling behavior, crystallinity, and material degradation [27]. The DD% was calculated from FTIR and found to be 94 ± 1.25% for chitosan synthesized from* U. duvauceli* which is comparatively higher than Std.CS (~85%). The DD% was higher than the result reported on chitosan isolated from the gladius of* Sepioteuthis lessoniana* (87.93%) [34] and comparable with gladius of* Todarodes pacificus* (96.2%) [35], since the DD% values may increase with increase in NaOH concentration and deacetylation time and also depend upon the source.

X-ray diffraction was used to detect the crystallinity and crystallite sizes of CSNA and Std.CS were shown in Figure 2(b). Both the spectra show a broad diffraction peak at 20° was assigned to be the prominent diffraction peak of (110) chitosan [36]. The peak intensity of CSNA was found to be higher than Std.CS representing higher crystallinity of CSNA than standard sample. The relative crystallinity index (CI) of CSNA and Std.CS was calculated using the following equation:

(5) $$\begin{array}{c} \text{Crystallinity Index}\left(\;CI\%\;\right)=\frac{\left(I_{110}-I_{am}\right)}{I_{110}}\times 100, \end{array}$$

where *I*_110_ (arbitrary units) is the maximum intensity of the (110) peak, which is usually around 2*θ* = 20°, and *I*_am_ (arbitrary units), which is the amorphous diffraction at 2*θ* = 16°. From this, the crystallinity index of Std.CS is 64.75%, CSNA with superior crystallinity of 85.47%. The apparent crystallite size *D*_app_(110) of CSNA in the direction perpendicular to the (110) crystal plane was calculated with the aid of the Scherrer equation:

(6) $$\begin{array}{c} D_{app}\left(\;110\;\right)=\frac{k\lambda }{\beta \left(\cos\left(\theta \right)\right)}, \end{array}$$

where *β* is the half-width of the reflection corrected for instrumental broadening (in radians); *k* is constant, indicative of crystallite perfection, which was assumed to be 0.9; and *λ* is the wavelength of used X-ray radiation. The apparent crystallite size of CSNA was calculated to be ~2.8 nm which was in well agreement with those crystallites highlighted in the HRTEM image of CSNA (Figure 1(b)).

**Figure 3:**
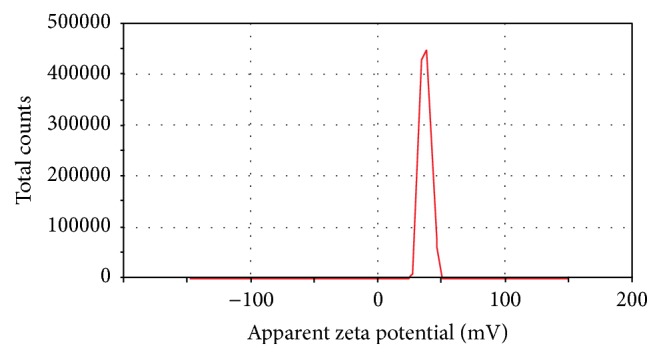
Zeta potential of CSNA synthesized from gladius of squid.

Zeta potential (Zp) of CSNA possesses mean positive potential of 38.5 ± 1.25 mV, ranging from +10 to +60 mV under acidic condition (Figure 3). The surface charge potential of CSNA was found to be physically stable, which determines the biological properties of biopolymer. The zeta potential was highly comparable with previous reported result [37].

The molecular weight determined was found to be 109.35 kDa for CSNA synthesized from gladius of* U. duvauceli*. This low molecular weight may be due to the source of chitosan and also varies with other parameters such as intermolecular hydrogen bonding, dissolved oxygen concentration, chitin concentration, particle size, reaction time, concentration of alkali, and high temperature [35]. Additionally, the ash content and moisture content for CSNA analyzed were 0.3 ± 0.05% and 0.62 ± 0.15% comparable to Std.CS (0.57% ash and 0.93% moisture content). The physical properties such as water and fat binding capacity of CSNA were 569.3 ± 1.25% and 324.2 ± 2.25% whereas commercial chitosan was 509 ± 0.25% and 298 ± 1.05%, respectively. The chitosan nanoaggregates showed high water absorbance which reveals the property of higher hygroscopicity nature [31, 38].

### 3.2. Antimicrobial Activity

To compare the antimicrobial activity of CSNA and Std.CS, the samples were tested against various pathogenic microorganisms and the results were listed in Table 1. From the results, both the chitosan samples showed antimicrobial property due to the presence of amino groups. Highest level of zone of inhibition was found in gram positive bacteria,* Staphylococcus aureus* (22 mm), followed by gram negative bacteria* Escherichia coli* (20 mm) for chitosan nanoaggregates at 1.0 mg/mL concentration. In case of fungal organisms, chitosan nanoaggregates showed high activity for* Aspergillus niger* with 19 mm, zone of inhibition. In case of Std.CS, significant result was shown against* E. coli* (18 mm) and few other microorganisms, which was evident with another report [39]. This was in agreement with the various reports on antimicrobial efficiency of chitosan with different molecular weight which showed high activity against* S. aureus* compared to* E. coli* may due to increased cellular uptake [15–17]. The MIC was tested against low concentrations of samples. The results revealed that the MIC against* S. aureus *and* E. coli* was lower than the tested microorganisms, whereas highest MIC was recorded against* C. albicans*. Moreover, the degree of antimicrobial effects of tested samples was higher with increase in concentration. Better antimicrobial activity of CSNA may be due to the interaction mechanism between nanoaggregates and the cell wall [40], in which the positively charged CSNA molecule and negative charged microbial cell wall constituents will interact and interrupt the normal cell mechanism related to DNA and thus inhibit RNA synthesis [41]. These results were consistent with the previous finding of nanosized chitosan against* E. coli* for potential application as food packing antimicrobial films [16]. Moreover, the antimicrobial property of chitosan showed strong bactericidal effect which may probably associated with molecular weight, degree of deacetylation, concentration of chitosan, and bacterial inoculum size [38, 42, 43].

### 3.3. Antioxidant Potential of CSNA

**Figure 4:**
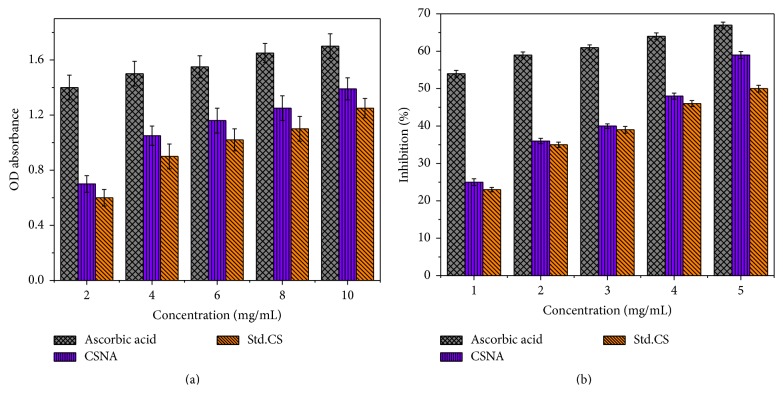
(a) Antioxidant reducing power and (b) superoxide radical scavenging ability of CSNA and Std.CS.

In order to study the antioxidant activity of CSNA and Std.CS, different concentrations of samples were prepared and analyzed for reducing power and scavenging property. All the samples showed highly effective reducing power as shown in Figure 4(a). The CSNA with 10 mg/mL concentration showed high reducing power of 1.39. When compared to Std.CS (1.25 for 10 mg/mL), the nanoaggregate of chitosan from gladius of squid showed high reducing power values. Reducing power of CSNA correlated with the concentration of sample; that is, increase in concentration will increase the absorbance which indicates higher reducing power and vice versa. The reducing ability of CSNA may vary with the degree of deacetylation, deacetylation times, and molecular weight [38].  Figure 4(b) shows concentration dependent scavenging activity against superoxide radicals. At 1–5 mg/mL concentration, the scavenging percentage of CSNA against superoxide radical ranged from 25 to 59% which was found to be higher than Std.CS (23–50%). These depict the concentration dependent scavenging effect of chitosan derivatives for superoxide anion radicals. The scavenging ability of Std.CS was comparable with the reported results [9], whereas the CSNA showed higher activity than its normal. These results revealed good reducing power and scavenging ability promising an excellent source of antioxidant supplement.

### 3.4. *In Vitro* Cell Toxicity Assessment

**Figure 5:**
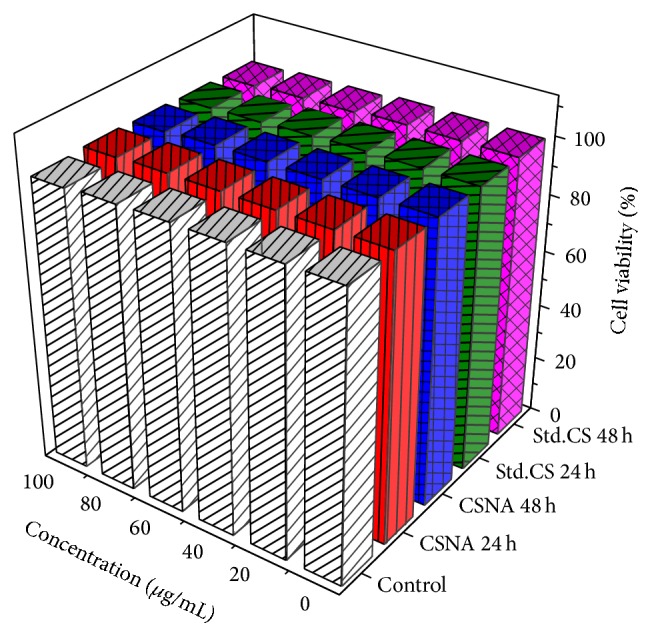
*In vitro* cell proliferation of HeLa against CSNA and Std.CS (24 h and 48 h) measured using MTT assay.

The cytotoxicity of CSNA and Std.CS was analyzed by MTT assay which is based on the conversion of MTT to formazan crystals by mitochondrial dehydrogenases (Figure 5). Surprisingly, the results did not evidence significant effect on cell viability. The cell shows a viability of over 99% during the experiments with those concentrations of nanoaggregates when compared with the negative control. The highest concentration of nanoaggregates evidenced 99.86% of cellular viability. Similarly, the commercial chitosan was also found to be less toxic (98.52%) towards the cell line. Moreover, up to 60 *μ*g/mL concentration the chitosan nanoaggregates were found to induce cell proliferation in 24 h of incubation. No significant differences in cell viability were observed among CSNA concentrations [19]. However, the low cytotoxic CSNA against HeLa cell were consistent with the previous finding for the application of siRNA delivery [24, 44]. Chitosan was acknowledged as more suitable for drug delivery across epithelial membrane based on the favourable biological characteristics [45, 46]. Several studies have demonstrated that the nanoparticles are transported across cell membrane more rapidly than microparticles [37, 47]. CSNA can be easily prepared under mild conditions and it can easily incorporate with biological macromolecules. In addition, the less toxic CSNA were reported as potential edible food packaging films [12]. These findings indicate that the chitosan nanoaggregates with low toxicity could be a potential edible drug delivery agent for cancer therapy.

## 4. Conclusions

Chitosan nanoaggregates were successfully synthesized by ionic gelation technique from the gladius of squid,* U. duvauceli*. The physiochemical properties reveal high degree of deacetylation, stable positive zeta potential, and low molecular weight along with very low ash content and high water binding capacity. CSNA also showed significant antioxidant reducing power and scavenging ability against superoxide radicals. Moreover, the excellent antimicrobial ability proposes that the CSNA can be used to control, suppress, or inhibit the growth of bacterial and fungal organisms. The nanoaggregates exhibited low toxicity in contact with HeLa cell line, which is an encouraging indicator of their cytocompatibility and safety. Therefore, the gladius thrown as waste could be utilized for the production of chitosan nanoaggregates for potential food packing film and biomedical application especially for drug delivery and in context of tissue engineering strategies.

## Figures and Tables

**Table 1:** Antimicrobial activity of CSNA and Std.CS against various microorganisms.

Microorganisms^a^	CSNA			Std.CS		
	Concentration (mg/mL)					
	0.25	0.50	1.0	0.25	0.50	1.0
*Micrococcus *sp.	5 ± 1.2	7 ± 0.6	12 ± 1.0	3 ± 1.0	5 ± 0.5	10 ± 1.5
*Staphylococcus aureus*	11 ± 1.5	17 ± 1.2	22 ± 2.5	7 ± 1.2	11 ± 2.0	16 ± 2.0
*Streptococcus *sp.	6 ± 1.0	8 ± 1.5	14 ± 0.5	2 ± 0.0	3 ± 0.0	5 ± 0.5
*Enterobacter *sp.	2 ± 0.6	5 ± 0.5	9 ± 1.5	1 ± 0.0	1 ± 0.0	3 ± 0.75
*Escherichia coli*	10 ± 1.5	16 ± 1.0	20 ± 2.5	6 ± 0.8	10 ± 1.2	18 ± 1.0
*Proteus vulgaris*	4 ± 2.0	6 ± 2.5	11 ± 1.0	0 ± 0.0	5 ± 1.05	8 ± 2.5
*Aspergillus niger*	10 ± 0.5	14 ± 0.45	19 ± 1.05	4 ± 0.5	7 ± 0.25	11 ± 0.45
*Rhizopus *sp.	4 ± 1.0	7 ± 2.5	10 ± 2.2	2 ± 1.5	4 ± 1.4	9 ± 2.0
*Candida *sp.	3 ± 0.25	5 ± 1.50	7 ± 1.0	2 ± 0.0	3 ± 0.0	4 ± 0.0

^a^DMSO was used as control and showed no inhibition zone.

## References

[1] Younes I., Hajji S., Frachet V., Rinaudo M., Jellouli K., Nasri M. (2014). Chitin extraction from shrimp shell using enzymatic treatment. Antitumor, antioxidant and antimicrobial activities of chitosan. *International Journal of Biological Macromolecules*.

[2] Manni L., Ghorbel-Bellaaj O., Jellouli K., Younes I., Nasri M. (2010). Extraction and characterization of chitin, chitosan, and protein hydrolysates prepared from shrimp waste by treatment with crude protease from *Bacillus cereus* SV1. *Applied Biochemistry and Biotechnology*.

[3] Youn D. K., No H. K., Prinyawiwatkul W. (2013). Preparation and characteristics of squid pen *β*-chitin prepared under optimal deproteinisation and demineralisation condition. *International Journal of Food Science and Technology*.

[4] Zamani A., Taherzadeh M. J. (2012). Production of superabsorbents from fungal chitosan. *Iranian Polymer Journal*.

[5] Mogilevskaya E. L., Akopova T. A., Zelenetskii A. N., Ozerin A. N. (2006). The crystal structure of chitin and chitosan. *Polymer Science—Series A*.

[6] Chen C.-H., Wang F.-Y., Ou Z.-P. (2004). Deacetylation of *β*-chitin. I. Influence of the deacetylation conditions. *Journal of Applied Polymer Science*.

[7] Chandumpai A., Singhpibulporn N., Faroongsarng D., Sornprasit P. (2004). Preparation and physico-chemical characterization of chitin and chitosan from the pens of the squid species, *Loligo lessoniana* and *Loligo formosana*. *Carbohydrate Polymers*.

[8] Mundargi R. C., Babu V. R., Rangaswamy V., Patel P., Aminabhavi T. M. (2008). Nano/micro technologies for delivering macromolecular therapeutics using poly(d,l-lactide-co-glycolide) and its derivatives. *Journal of Controlled Release*.

[9] Subhapradha N., Ramasamy P., Shanmugam V., Madeswaran P., Srinivasan A., Shanmugam A. (2013). Physicochemical characterisation of *β*-chitosan from *Sepioteuthis lessoniana* gladius. *Food Chemistry*.

[10] Anusha J. R., Justin Raj C., Cho B.-B., Fleming A. T., Yu K.-H., Kim B.-C. (2015). Amperometric glucose biosensor based on glucose oxidase immobilized over chitosan nanoparticles from gladius of *Uroteuthis duvauceli*. *Sensors and Actuators B: Chemical*.

[11] Agnihotri S. A., Mallikarjuna N. N., Aminabhavi T. M. (2004). Recent advances on chitosan-based micro- and nanoparticles in drug delivery. *Journal of Controlled Release*.

[12] Schreiber S. B., Bozell J. J., Hayes D. G., Zivanovic S. (2013). Introduction of primary antioxidant activity to chitosan for application as a multifunctional food packaging material. *Food Hydrocolloids*.

[13] Anusha J. R., Fleming A. T., Kim H.-J., Kim B. C., Yu K.-H., Raj C. J. (2015). Effective immobilization of glucose oxidase on chitosan submicron particles from gladius of *Todarodes pacificus* for glucose sensing. *Bioelectrochemistry*.

[14] Babu V. R., Patel P., Mundargi R. C., Rangaswamy V., Aminabhavi T. M. (2008). Developments in polymeric devices for oral insulin delivery. *Expert Opinion on Drug Delivery*.

[15] Dutta P. K., Tripathi S., Mehrotra G. K., Dutta J. (2009). Perspectives for chitosan based antimicrobial films in food applications. *Food Chemistry*.

[16] Romainor A. N. B., Chin S. F., Pang S. C., Bilung L. M. (2014). Preparation and characterization of chitosan nanoparticles-doped cellulose films with antimicrobial property. *Journal of Nanomaterials*.

[17] Sadeghi-Kiakhani M., Arami M., Gharanjig K. (2013). Application of a biopolymer chitosan-poly(propylene)imine dendrimer hybrid as an antimicrobial agent on the wool fabrics. *Iranian Polymer Journal (English Edition)*.

[18] Adibzadeh S., Bazgir S., Katbab A. A. (2014). Fabrication and characterization of chitosan/poly(vinyl alcohol) electrospun nanofibrous membranes containing silver nanoparticles for antibacterial water filtration. *Iranian Polymer Journal*.

[19] Grenha A., Gomes M. E., Rodrigues M. (2010). Development of new chitosan/carrageenan nanoparticles for drug delivery applications. *Journal of Biomedical Materials Research—Part A*.

[20] Konecsni K., Low N. H., Nickerson M. T. (2012). Chitosan-tripolyphosphate submicron particles as the carrier of entrapped rutin. *Food Chemistry*.

[21] Li G.-F., Wang J.-C., Feng X.-M., Liu Z.-D., Jiang C.-Y., Yang J.-D. (2015). Preparation and testing of quaternized chitosan nanoparticles as gene delivery vehicles. *Applied Biochemistry and Biotechnology*.

[22] Sudheesh Kumar P. T., Lakshmanan V.-K., Anilkumar T. V. (2012). Flexible and microporous chitosan hydrogel/nano ZnO composite bandages for wound dressing: *in vitro* and *in vivo* evaluation. *ACS Applied Materials & Interfaces*.

[23] Roney C., Kulkarni P., Arora V. (2005). Targeted nanoparticles for drug delivery through the blood-brain barrier for Alzheimer's disease. *Journal of Controlled Release*.

[24] Lee D. W., Yun K.-S., Ban H.-S., Choe W., Lee S. K., Lee K. Y. (2009). Preparation and characterization of chitosan/polyguluronate nanoparticles for siRNA delivery. *Journal of Controlled Release*.

[25] Jang M.-K., Kong B.-G., Jeong Y.-I., Lee C. H., Nah J.-W. (2004). Physicochemical characterization of *α*-chitin, *β*-chitin, and *γ*-chitin separated from natural resources. *Journal of Polymer Science Part A: Polymer Chemistry*.

[26] Huang K.-S., Sheu Y.-R., Chao I.-C. (2009). Preparation and properties of nanochitosan. *Polymer—Plastics Technology and Engineering*.

[27] Baxter A., Dillon M., Anthony Taylor K. D., Roberts G. A. F. (1992). Improved method for i.r. determination of the degree of N-acetylation of chitosan. *International Journal of Biological Macromolecules*.

[28] AOAC (1984). *Official Methods of Analysis of the Association of Official Analytical Chemistry*.

[29] Oyaizu M. (1986). Studies on products of browning reactions: antioxidative activities of products of browning reaction prepared from glucosamine. *Japanese Journal of Nutrition*.

[30] Jing T. Y., Zhao X. Y. (1995). The improved pyrogallol method by using terminating agent for superoxide dismutase measurement. *Progress in Inorganic Biochemistry and Biophyscis*.

[31] Abdou E. S., Nagy K. S. A., Elsabee M. Z. (2008). Extraction and characterization of chitin and chitosan from local sources. *Bioresource Technology*.

[32] Huang J., Chen W.-W., Hu S. (2013). Biochemical activities of 6-carboxy *β*-chitin derived from squid pens. *Carbohydrate Polymers*.

[33] Rampino A., Borgogna M., Blasi P., Bellich B., Cesàro A. (2013). Chitosan nanoparticles: preparation, size evolution and stability. *International Journal of Pharmaceutics*.

[34] Subhapradha N., Saravanan R., Ramasamy P., Srinivasan A., Shanmugam V., Shanmugam A. (2014). Hepatoprotective effect of *β*-chitosan from gladius of *Sepioteuthis lessoniana* against carbon tetrachloride-induced oxidative stress in wistar rats. *Applied Biochemistry and Biotechnology*.

[35] Youn D. K., No H. K., Prinyawiwatkul W. (2013). Preparation and characterisation of selected physicochemical and functional properties of *β*-chitosans from squid pen. *International Journal of Food Science and Technology*.

[36] Vasconcellos F. C., Goulart G. A. S., Beppu M. M. (2011). Production and characterization of chitosan microparticles containing papain for controlled release applications. *Powder Technology*.

[37] Liu H., Gao C. (2009). Preparation and properties of ionically cross-linked chitosan nanoparticles. *Polymers for Advanced Technologies*.

[38] Arancibia M. Y., Alemán A., Calvo M. M., López-Caballero M. E., Montero P., Gómez-Guillén M. C. (2014). Antimicrobial and antioxidant chitosan solutions enriched with active shrimp (*Litopenaeus vannamei*) waste materials. *Food Hydrocolloids*.

[39] Celis D., Azocar M. I., Enrione J., Paez M., Matiacevich S. (2011). Characterization of salmon gelatin based film on antimicrobial properties of chitosan against *E. coli*. *Procedia Food Science*.

[40] Azadi G., Seward M., Larsen M. U., Shapley N. C., Tripathi A. (2012). Improved antimicrobial potency through synergistic action of chitosan microparticles and low electric field. *Applied Biochemistry and Biotechnology*.

[41] Saita K., Nagaoka S., Shirosaki T., Horikawa M., Matsuda S., Ihara H. (2012). Preparation and characterization of dispersible chitosan particles with borate crosslinking and their antimicrobial and antifungal activity. *Carbohydrate Research*.

[42] Chen C.-P., Chen C.-T., Tsai T. (2012). Chitosan nanoparticles for antimicrobial photodynamic inactivation: characterization and in vitro investigation. *Photochemistry and Photobiology*.

[43] Friedman A. J., Phan J., Schairer D. O. (2013). Antimicrobial and anti-inflammatory activity of chitosan-alginate nanoparticles: a targeted therapy for cutaneous pathogens. *Journal of Investigative Dermatology*.

[44] Rudzinski W. E., Aminabhavi T. M. (2010). Chitosan as a carrier for targeted delivery of small interfering RNA. *International Journal of Pharmaceutics*.

[45] Honary S., Zahir F. (2013). Effect of zeta potential on the properties of nano-drug delivery systems—a review (part 1). *Tropical Journal of Pharmaceutical Research*.

[46] Soppimath K. S., Aminabhavi T. M., Kulkarni A. R., Rudzinski W. E. (2001). Biodegradable polymeric nanoparticles as drug delivery devices. *Journal of Controlled Release*.

[47] Thakker S. P., Rokhade A. P., Abbigerimath S. S. (2014). Inter-polymer complex microspheres of chitosan and cellulose acetate phthalate for oral delivery of 5-fluorouracil. *Polymer Bulletin*.

